# Understanding an Urban Park through Big Data

**DOI:** 10.3390/ijerph16203816

**Published:** 2019-10-10

**Authors:** Jisoo Sim, Patrick Miller

**Affiliations:** Landscape Architecture Program, College of Architecture and Urban Studies, Virginia Tech, 800 Drillfield Dr., Blacksburg, VA 24060, USA; pmiller@vt.edu

**Keywords:** urban park, onsite survey, user analysis, big data, social media analytics, sentiment analysis

## Abstract

To meet the needs of park users, planners and designers must know what park users want to do and how they want the park to offer different activities. Big data may help planners and designers gain this knowledge. This study examines how big data collected in an urban park could be used to identify meaningful implications for planning and design. While big data have emerged as a new data source, big data have not become an accepted source of data due to a lack of understanding of big data analytics. By comparing a survey as a traditional data source with big data, this study identifies the strengths and weaknesses of using big data analytics in park planning and design. There are two research questions: (1) what activities do park users want; and (2) how satisfied are users with different activities. The Gyeongui Line Forest Park, which was built on an abandoned railway, was selected as the study site. A total of 177 responses were collected through the onsite survey, and 3703 tweets mentioning the park were collected from Twitter. Results from the survey show that ordinary activities such as walking and taking a rest in the park were the most common. These findings also support existing studies. The results from social media analytics found notable things such as positive tweets about how the railway was turned into a park, and negative tweets about diseases that may occur in the park. Therefore, a survey as traditional data and social media analytics as big data can be complementary methods for the design and planning process.

## 1. Introduction

Although big data have emerged as a critical source of data and are playing an essential role in urban studies, it is still uncommon in park planning and design. Understanding urban settings by using big data can reveal heretofore hidden characteristics of urban areas [1,2,3]. The use of social media data, a type of big data, has the potential to provide a deeper understanding of human attitudes and perceptions toward urban places [4] and explain resulting behaviors [5,6]. There are significant strengths of social media data such as indisputability, volume, and real-time data. Since social media users post their opinions on their social media platform [7], social media data represent public opinions more directly than other traditional methodologies when researchers try to understand public attitudes and perceptions. Social media can generate enormous amounts of data. In the case of Twitter, over 473,400 tweets are posted every minute around the world, and 2.5 quintillion bytes of data are created every single day [8]. Social media data can also be collected in real-time [9]. While other researchers have been attuned to the advantages of big data in social science research, researchers in landscape architecture and urban planning rarely use big data analytics.

One study used social media data to identify successful public spaces [10], and another one used location-based data to trace the human visit dynamics in parks [11]. However, due to the difficulties in measuring social media data [12] and concern over how closely the content analysis can link to that context [13,14], different content analysis such as sentiment and frequency analysis are rarely used.

The approach used most often in the past, or the traditional approach, for designing and planning open spaces is called “the demand approach” [15]. The demand approach uses the stated desires of people from interviews or questionnaires and provides recommendations of recreation and amenities to meet those demands. As stated above, most parks, urban forests, and greenways are planned and designed using the demand approach [16,17,18,19,20]. If done properly, these methods require considerable time and expense to collect the needed data.

One of the reasons for the reluctance in using social media data is that the reliability of social media analytics has not been verified in the design and planning. Therefore, this study examined how social media data can be used to understand: (1) visitor activities in parks and (2) visitor satisfaction about the park. This was done by comparing two different methods: (1) a survey as traditional data and (2) social media analytics as big data. The survey will examine how people indicate they use a park, then collected tweets mentioning the park will be analyzed. After that, the results derived from the two approaches will be compared to identify the similarities and differences between the two methods.

This article contains five sections: introduction, literature review, methods, results, and conclusion. The literature review answers the benefits that park visits provide to users, how to assess user activities and their attitudes, and the pros and cons of the survey and social media data. In the methods section, we address the study site, data collection, and analytic methods used. The results section provides our findings. We then discuss the conclusions and implications in the conclusions.

## 2. Literature Review

This section describes how urban parks are used and compares two different methods that can be used to understand how people use urban parks. Parks benefits can be grouped into three categories: economic, health, and environmental benefits [21,22]. This section describes the social benefits of urban parks and how urban parks contribute to social interactions. Then, we discuss traditional analytics including surveys and big data analytics focused on social media analytics in urban studies. After that, we compare the pros and cons of the two methods.

### 2.1. Social Benefits and Social Interaction in Urban Parks

Several terms have been used in studies of the social benefits from urban parks. Many studies have investigated the role of urban parks as an essential space to increase the quality of daily life of an urbanized society [23,24]. Empirical evidence supports that urban parks, greenways, and urban forests in urban contexts improve the quality of life in many ways. In addition to environmental benefits such as pollution purification, urban parks contribute to improving health, enhancing social interactions, and providing peacefulness [25,26,27,28]. Social benefit is a crucial metric to determine the success of a redevelopment project. Social benefit is related to how the project generates urban vitality. Urban vitality can be measured by social interactions [29,30,31,32]. Social interaction, as a process of interactivity among more than two people and the relationship between people and spaces, includes all forms of communication such as cooperation, competition, playing, informing, negotiating and bargaining, and creates the placeness [30,31,32]. Furthermore, urban vitality can be considered in various ways. Jane Jacobs describes urban vitality as a place to provide chances for good relations between people [33]. Jalaladdini and Oktay (2012) states that urban vitality is a safer, more desirable, and more attractive place that offers more choices for social activities [34]).

Ulrich [27], for example, compares psychophysiological reactions toward three landscape types: landscapes with vegetation, with water, and with urban content. By using metrics that represent physiological and psychological measures, he found that vegetation and water landscapes had greater beneficial influences on the positive psychological feelings of park users. Chiesura [26] states that urban parks provide social and psychological benefits and enrich our lives with meaning and emotions. She explains that being in nature makes people feel positive. To do a better job of planning and designing urban parks that will offer positive emotions, we need to understand the tools or approaches that can be used. These include the role of traditional methods and big data approaches.

Peters, Elands, and Buijs [35] assert that urban parks can be a trigger to generate social interactions by stimulating social cohesion. They used a survey, observations, and interviews to carry out social interaction research in five urban parks in the Netherlands. According to this study, urban parks can promote the mingling of different ethnic groups and encourage interactions among visitors. Coley, Sullivan, and Kuo [36] verified that urban parks encouraged social interactions among residents and contributed to social cohesion. Their findings revealed that natural elements increased opportunities for social interactions, and using outdoor places promoted communication within neighborhoods [36]. Natural environments can also help people relax as well as promote social interactions [37]. Kuo et al. [37] showed that the presence of greenspaces in the inner city generated positive responses from residents. The density of urban nature may also increase the sense of safety. These studies show practical evidence that urban parks not only foster social interaction, but also contribute to the sense of safety in urban environments. These studies show the practical implications that urban parks contribute to urban vitality by fostering social interactions.

Age groups have been considered as an important predictor of park visits, especially for people aged over 50 years who visit parks and participate in park activities at a lower rate than other age groups [38,39,40]. Numerous studies have stated that physical activities and visits to a park significantly decrease with age. Another sociodemographic characteristic is gender. In 1997, Portes [41] indicated that gender represents a major dimension of social structure and offers important insights to understand many phenomena. In more recent research, Reed, Price, Grost, and Mantinan [42] found significant differences in gender and park use. Social media data can answer questions by detecting social networks revealed on a social media platform [43]. Social network analysis contributes to detecting communities [44], social roles, and social cohesion [43].

### 2.2. Traditional Analytics for Understanding People

There are several methods to understand how people use a place and how people interact with each other in a place. Researchers have developed several ways to measure these variables through field observation, surveys, and interviews. Since these methods have been used in studies of urban parks for a long time, these methods are often called traditional analytics [45,46]. Some studies have termed these methods as small data analytics when compared to new methods such as big data analytics [47]. In this study, we call these methods traditional analytics to stress their historicity.

Among the traditional analytics such as an interviews, observations, and focus group meetings, surveys can be considered as the representative method for studying urban parks and behaviors [48,49]. A survey can satisfy two main concerns of traditional methods: fairness and efficiency. As many surveys are targeted to anticipated park users and often obtain data about the survey participants, it is easy to verify who the participants are and the extent to which they are representative of park users. In many studies, a survey is used to understand people’s attitudes and their behavior in urban open spaces such as parks. Peters, Elands and Buijs [35] used a survey to find that the urban parks can promote social cohesion. Whiting, Larson, Green, and Kralowec [50] used a park visitors survey to identify motivation and preferences for outdoor recreation.

While traditional assessment tools can be used to evaluate the on-site benefits of parks through preset surveys, interviews, and observations [51,52,53,54], the weakness of these methods is that the intention of the researcher can be reflected in the questions asked and in the wording of the questions. One limitation of many surveys is that they are self-selecting. Participation is voluntary. Those who are motivated to participate may not represent all park users. Another limitation is that surveys require the researcher put the questionnaire together to preconceive what types of activities users may want to participate in. If certain activities are not included in the survey, they will not appear in the results. A survey can provide good data when it includes activities of interest to the participants. How a survey compares to big data analytics will be discussed next.

### 2.3. Big Data Analytics as New Techniques for Understanding Park Usage

Big data sources, especially social media data, hold the potential for enhancing the understanding of human behaviors and perceptions of urban places. Social media such as Facebook, Twitter, Instagram, and Flickr are widely used by people to post and share their opinions and communicate with each other [7]. Social media can be considered important source data to identify and understand how people interact within urban spaces. Although accessing social media data is not free, and researchers often have to pay fees for the data or licensing agreement, it still has considerable strengths. There are three reasons as to why social media could be a valuable data source: (1) social media users post text to express their thoughts directly [55]; (2) collecting social media data allows researchers to trace the past data, and (3) social media data are cheap data and their volume is enormous when compared to traditional data. According to social cognitive theory (SCT), it explains how people memorize in terms of three things: (1) their understanding of the activities; (2) their participation; and (3) the physical environmental [56]. Users post information on social media to share their thoughts and communicate with each other [7]. When we considered the motivation of using social media based on SCT, there are two kinds of factors: intrinsic (personal) and extrinsic (environmental) factors. In terms of the extrinsic factors, user behavior is affected by people sharing information [7]. Big data, especially social media data, may be considered as representative media to capture a user’s thoughts and behaviors.

Sentiment analysis is one way to evaluate one’s perceptions and emotions, whether they are positive or negative [57,58,59,60] by using social media data. Among the sentiment analysis classification, the lexicon-based approach was used in this study. The lexicon-based approach uses positive and negative sentiment terms by using a dictionary based-approach [61]. The algorithm gives one point to the positive words and subtracts one point for every negative word. Then, all scores are evaluated for total content. The algorithm gives a zero score to content that has no positive (higher than 0) or negative words (lower than 0) or offsets positive and negative words in the content. The score refers to the users’ attitude toward a park. The sentiment score derived from the algorithm shows whether the users were satisfied with their experience of the park. If the score is bigger than zero, the score is counted as a positive experience. If the score is under zero, it represents a negative experience.

Although big data are accepted as reliable data, there are several challenges that need to be overcome when using big data. First, big data require a researcher to have specific computer skills to access and analyze the data. To write a code that uses software packages requires that a researcher is knowledgeable about computer program languages such as R, Python, Java, and C [62]. Second, it is common for some researchers to believe that big data are available to all without the need to pay a fee [63]. Accessing big data sources is not free. Social media data from Twitter, and Facebook require the terms of services (ToS) to be continuously updated and changed to protect privacy issues [9]. Compared to big data, traditional data sources such as census data are more freely and widely accessible and more available for everyone to analyze with standard software. Third, there is a lack of standardization of methods for collecting and analyzing big data. While some studies very precisely address data collection and methods [64], other studies are rather vague [65,66]. Fourth, big data analytics will contain sampling errors. In the case of traditional methods such as a survey or interview, there are standard methods to minimize the sampling error [67,68]. However, big data, especially social media data, have challenges beyond data collection [69]. Users who post on social media are not likely to be representative of all users. While social media may provide meaningful implications about people and their behavior in an urban setting [9,70] in terms of its volume, social media data also have limitations that it may be biased from the opinions of younger people [69]. Hargittai (2015) pointed out that big data studies have methodological challenges of limited sampling frames from those inclined to use social media platforms. For example, Facebook users are younger, and the results from that platform tend to reflect younger opinions [71].

### 2.4. Comparison of Traditional Methods with Big Data Analytics

Survey data as a traditional method of collecting data are strong as the sampling process can be easier when compared to big data analytics. In terms of a sample, an onsite survey may select a visitor by contacting every fifth or tenth visitor for validity in a sampling process [67,68]. However, since researchers cannot identify the demographic information of social media users, it is difficult to control sampling validity in big data analytics [69].

Compared to traditional data, social media data provide valuable information about the behavior of people in a specific space. While social media data have limitations related to a lack of generalization to the entire population [72], social media data may have other advantages. First, there is an enormous number of users around the world who publish their daily activities. Second, social media data include a variety of types of information such as trivial travel experiences that cannot be documented with traditional methods. When we considered the motivation of using social media based on social cognitive theory, there are two types of factors: intrinsic (personal) and extrinsic (environmental) factors. In terms of the extrinsic factors, the users’ behaviors are affected by people sharing information [7].

## 3. Methodology

This section describes the study site, how data were collected, and the analytics used. The authors selected the Gyeongui Line Forest Park in Seoul, Korea, which has emerged as an urban hotspot to examine how visitors use the park through a survey and social media analysis. Since the park is surrounded by four universities, it has become a hotspot for young generations in Korea. For collecting data, a survey as a traditional method and social media as big data were selected. Among the traditional methods, a survey covers larger samples [67] and represents targeted information that is designed by researchers. Social media data also cover large samples and social media platforms are used to post and share the thoughts and daily activities of social media users [6]. Several studies have compared survey data and social media data [73,74]. This study selected a survey and social media data for comparison. At the park, an onsite survey was conducted on weekdays and weekends. Twitter postings that mentioned the park title were also collected during this period. With the survey and social media data, statistical analysis and sentiment analysis were conducted to derive results.

### 3.1. Study Site

**Figure 1:**
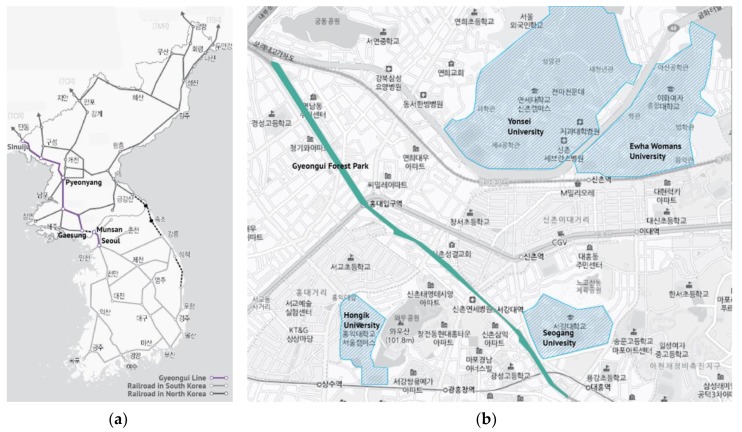
(**a**) Rail road system of Korea; (**b**) Gyeongui Line Forest Park.

As above-mentioned, the Gyeongui Line Forest Park was selected as the study site. This park was used as a railroad for the last 100 years (Figure 1a). When the railroad was turned into an underground railroad, the site was vacant and abandoned. As one of the urban revitalization projects, the Seoul Metropolitan Government built the park on the vacant land. The Seoul Metropolitan Government expected the park to facilitate the economic growth of adjacent areas [75]. From Mapo-gu to Yongsan-gu, Seoul, the park, 6.3 km in length, crosses the center of the city. The park was divided into three phases, and each phase was completed in 2012, 2015, and 2016, respectively [75].

At present, the park has become the center of younger generations because of its location. Four universities, Yonsei, Hongik, Seokang, and Ewha Women’s University are located within a 700 m radius from the park (Figure 1b). The park has emerged as an urban hotspot where young people gather and frequently visit [76]. Well-known districts for young people, Sinchon, Hongdae, and Ewha districts, are also located near the park.

### 3.2. Data Collection

The data were collected using two different methods: a survey and social media (Table 1). For the survey data, an onsite survey was conducted and a total of 192 samples were collected. For the social media data, postings mentioned in the keywords related to the park were collected and a total of 3703 tweets were filtered.

#### 3.2.1. Survey

The survey was used as the traditional method for comparison with social media data and to understand user activities in the park and user satisfaction about the park. Among the traditional methods, surveys can be considered as a way that satisfies two things: fairness and efficiency. Fairness is related to concepts such as democracy, representativeness, transparency, and public acceptability [77]. This concept concerns the perceptions of participants and the public and whether public participation has been conducted in a manner that accurately reflects the views of the target population. The concept of efficiency refers to the ease with which data can be collected [77].

The survey was conducted from August 15–18 and October 1–8, 2018. The sampling dates at the park were selected to include weekdays and weekends. At the park, the researcher approached every third adult visitor and asked of their willingness to take the park survey. A total of 192 respondents stated they were willing to participate in the survey, and 15 were disqualified because they did not complete the survey.

The questionnaire was divided into five sub-parts: park visiting frequency and activities, satisfaction of the visit, social interactions, social cohesions, and respondent information. The first part included information about the visits to the park (frequency, duration, company) and activities visitors did in the park [78]. Nine activities in the park were carefully selected following the previous literature [24,78,79]. User satisfaction included questions about the visitors’ feelings toward the park features and their experience ranged from ‘1 = strongly disagree’ to ‘5 = strongly agree’. For the social interaction, participants were asked to answer several variables including feeling safe, participating in social programs in the park, and forming new relationships with others. Since social cohesion occurs based on trusting social relationships such as a sense of community [80], the social cohesion part of the survey asked about the nature of trusting relationships with those in their neighborhood. Following Perez et al. (2015), survey participants were asked to self-report about social cohesion factors among residents and their willingness to take action for the common good. Based on questions from the literature, the authors also added a question about the residents’ attitudes toward and willingness to make social relationships with other visitors of the park.

#### 3.2.2. Social Media Data

For the social media data, all tweets mentioned in the keywords related to the park were collected from July to September 2018 through Twitter API. Every tweet that mentioned one of three keywords, ‘Gyeongui Line’, ‘Gyeongui Line Forest Park’, and ‘Yeontral Park’ were scrawled. Three keywords were selected as the park has different names: some call it the ‘Gyeongui Line Forest Park’, others refer to it as ‘Gyeongui Line’, while the younger generation know the park as ‘Yeontral Park’. All tweets in the dataset were posted inside South Korea. A total of 3703 postings were collected over three months.

### 3.3. Data Analytics

#### 3.3.1. Survey Analytics

Data analytics was designed to answer two main research questions: (1) what activities in the park could be detected through the survey and (2) how many visitors were satisfied with their experiences in the park. For analysis, the authors mainly used R Studio, which is a free and open source tool and an integrated development environment (IDE) for the R language [81]. Given its accessibility and the ease in using the R programming language, it was decided in this paper to use R.

For the survey data, descriptive statistics and correlation analysis were conducted to identify how visitors used the park and how satisfied they were with their park experiences. The surveyed data collected included user activities, user satisfaction, and social interaction. A frequency analysis was also conducted. After the frequency analysis, the relationship between satisfaction and social interactions was analyzed by using correlation analysis.

#### 3.3.2. Social Media Analytics

Regarding social media data, two main analytics were conducted to investigate the data. Text mining and sentiment analysis can be used to identify the implications. Text mining is usually divided into three areas: data mining, statistics, and linguistics, which aims to extract meaningful information from unstructured textual data [82]. The emergence of social media applications has contributed to the growth of text mining usage. We used Hu and Liu’s (2004) approach to conduct sentiment analysis [83]. Sentiment analysis was then used to understand the preferences and attitudes of users.

Frequency analysis was conducted to identify major words used in social media posts. Content analysis was then conducted on the posted texts to categorize them into single word categories. These categories were then counted to identify the main reason for the users’ sentiments. These sentiments can represent people’s opinions, sentiments, emotions, and attitudes [84]. This analysis provided the researchers with user assessments of the park and the reasons for those sentiments.

## 4. Results

The results were divided into three parts: the survey, social media, and a comparison of these two methods. The survey results indicate that visitors occasionally visited the park for relaxation. User satisfaction showed a positive relationship with social interaction. From the survey results, the park as an urban hotspot provides a place for relaxation and fosters social interaction and cohesion. The social media results revealed the positive attitudes of park visitors toward the park, especially attributes of the park such as ‘forest’ and ‘railroads’. The results of the social media analysis also pointed out that park visitors positively reacted to interactive activities with others. For the comparative test of the difference between the survey and social media, there were no differences between the satisfaction from the survey and positive postings from social media.

### 4.1. Survey Results

The survey data revealed the characteristics of the park visitors. Like in previous studies, the park tended to be visited by more females (54.24%) and young people under 40 years of age (64.41%). The majority of visitors came from other districts and around thirty percent of visitors were residents of adjacent areas to the park (Table 2).

The descriptive statistics of the survey explained the basic uses of the park. Table 3 provides an understanding of the visits. Many visitors came to the park less than once a month (29.9%) or were visiting the park for the first time (23.7%). Additionally, some portion of visitors visited the park for more than two days (14.1%) a week or every day (10.7%). This result supports that park visitors use the park occasionally rather than on a daily basis, and that visitors normally came to the park on special occasions rather than regular visits.

In terms of the stated desire for visiting and actual use of the park, Table 4 shows the results. Activities in four categories—physical activities, mental health, social interaction, and other activities—were asked and the results are stated in Table 4. Visitors came to the park to refresh their daily life (53.7%) and take a rest (36.2%). The main activity in this park differs slightly from existing studies that have identified physical activities as the main activities in a park [85]. The highest percentage of visitors used the park to interact with their friends or family (50.9%). Under 20 percent of visitors used this park to do physical activity. The average satisfaction score was based on a scale of 1 = rarely satisfied to 5 = highly satisfied. Users of the park were slightly satisfied (mean = 3.66), but the level of satisfaction was not that high. For social interaction in the park, users answered that the park contributed to reinforcing social interaction (mean = 3.56). The social cohesion satisfaction of adjacent community residents was 3.51. The satisfaction score was compared between two groups: visiting alone and visiting the park with friends or family. Pearson’s Chi-squared test was used to examine the relationship between factors where there was no difference between the two groups (*p*-value = 0.49). One of the results of the survey was the correlation analysis. The correlation between satisfaction and social interaction was positive (correlation coefficient = 0.61, *p*-value < 0.0001). Although many researchers have verified that social interaction is a social benefit from a park [32,35], the relationship between the park visitors’ satisfaction and social interaction level has rarely been identified.

**Figure 2:**
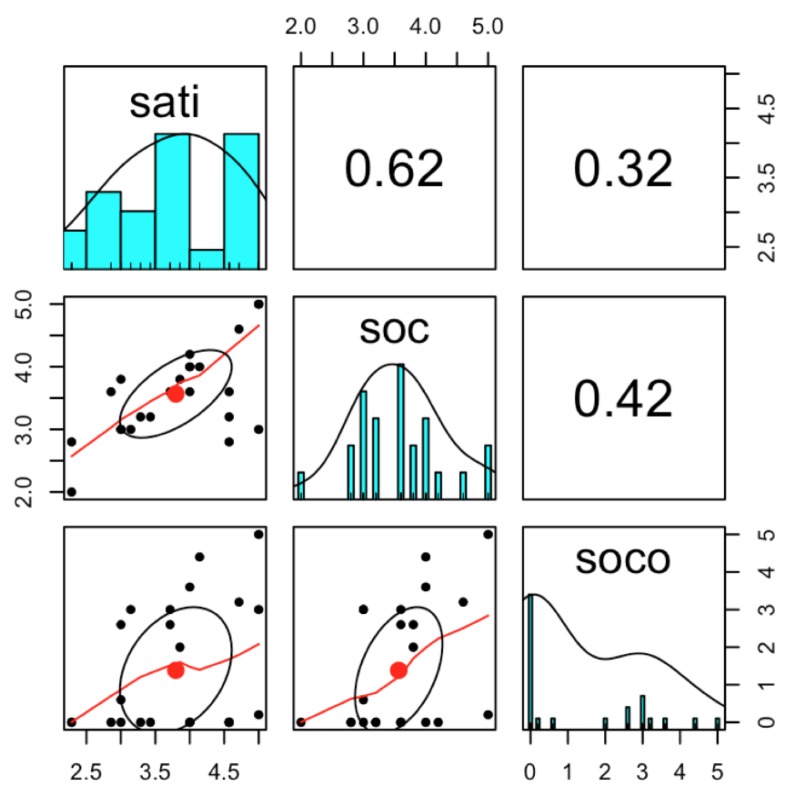
Correlation analysis between factors. Abbreviations: sati, satisfaction; soc, social interaction; soco, social cohesion.

Next, the correlation analysis was conducted once again to identify the relationship between satisfaction (sati), social interaction (soc), and social cohesion (soco) in the park (Figure 2). Figure 2 represents the correlation between these factors. The graphs with the red line refer to the correlation graphs between satisfaction (sati) and social interaction (soc), social interaction (soc) and social cohesion (soco), and satisfaction (sati) and social cohesion (soco). The numbers represent the correlation coefficient. The relationships between the factors of satisfaction, social interaction, and social cohesion were positive. For example, satisfaction and social interaction had a positive relationship (correlation coefficient = 0.62). There was also a positive relationship between satisfaction and social cohesion (0.32), but the relationship was not strong. Social interaction and social cohesion relationship also showed a positive relationship that was not strong (0.42). This means that visitors of the park who were satisfied with their experience tended to value social benefits such as increasing social interaction or social cohesion.

### 4.2. Social Media Results

**Figure 3:**
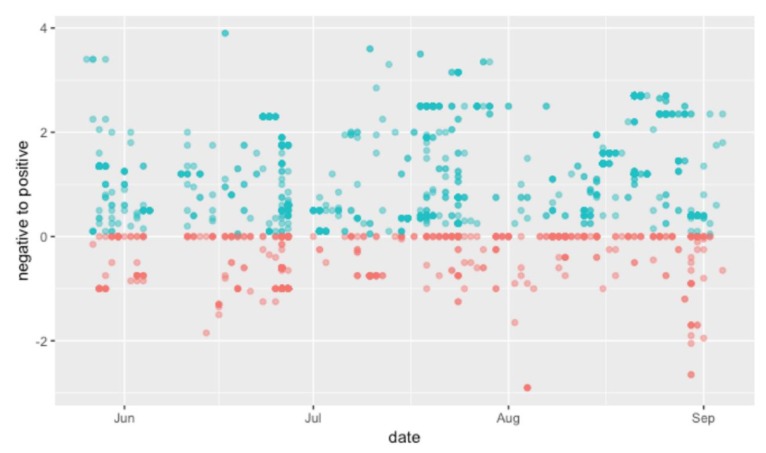
The result of the sentiment analysis (from negative sentiment (red, less than 0) to positive sentiment (blue, higher than 0)).

As mentioned in Section 2, the sentiment analysis of social media data provides insight into the users’ attitude toward the park. The results indicate that more users had a positive experience than those who had a negative one (Figure 3). Figure 3 shows the number of tweets and sentiment scores of each tweet during the study period. The results show that social media users generally reacted positively when they posted about the park. This also supports the previous study that social media users tend to highlight their positive attributes [86].

**Figure 4:**
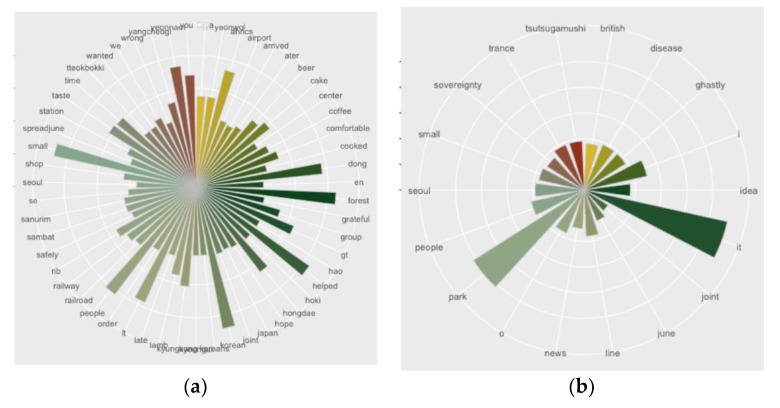
(**a**) Word frequency analysis of positive sentiments; (**b**) Word frequency analysis of negative sentiments.

To figure out the type of activities that made the positive or negative sentiment, the dataset divided into two sub-sets: the positive data, and the negative data. Then, the word frequency analysis was comprised to extract the main reasons for each sentiment. The most frequently mentioned words in the positive data were related to food and eating such as ‘beer’, ‘cake’, ‘coffee’, ‘cooked’, ‘lamb’, ‘tteokbokki (street-food in Korea)’, and ‘taste’. Physical features of the park were also mentioned in the dataset: ‘forest’, and ‘railroad’. This indicates that the unique character of the park is important to the positive experience of users. Words which implied the relationship between people were also mentioned frequently such as ‘people’, ‘we’, and ‘you’. (Figure 4a). From these results of the most frequently mentioned words, we can conclude that among the park visitors, social media users tended to have a positive experience about the unique attributes of the park such as ‘forest’ and ‘railroad’ and also tended to enjoy casual treats in the park. Furthermore, ’railroad’ and ‘railway’ refer to the previous railway on the grounded Gyeonggui Line.

The negative sentiments were more complex in real-life than the positive sentiments, since the result of the frequency analysis indicated that the number of users who mentioned a word was too low to draw any conclusions. In other words, each word was mentioned 20 times. Only some words such as ‘park’, ‘it’, ‘I’, and ‘people’ were mentioned over 20 times in the negative dataset. Other words that were mentioned between 10 to 20 times were ‘disease’, ‘tsutsugamushi (disease from lawn)’, and ‘ghastly’. All three words were related to the disease from the lawn. This data show that park visitors worried about the disease when they visited the park (Figure 4b).

In terms of activities in the park, social media data indicate that eating something with someone most often resulted in positive sentiment toward the park. This assumption was derived from the most frequently mentioned words such as ‘eating’, ‘foods’, and ‘we’. One of the more interesting results is the use of personal pronouns. When the users represented a negative sentiment, they used ‘I’ rather than ‘we’. From the use of pronouns, it seems that experiences with others tended to be positive while negative experiences tended to seen as relating to the individual. These insights from social media analytics could not be found in the survey results. Since the survey questionnaire was designed to identify participation in traditional activities in the park, the questionnaire did not capture the importance of park activities such as ‘eating’. However, social media data tended not to capture ordinary activities in the park such as ‘walking’ and ‘relaxing’. Perhaps this is because people take something like walking for granted. When people posted about their activities and experiences on Twitter, they did not upload ordinary, common activities of their daily life and preferred instead to share their more extraordinary or less common events with others.

### 4.3. Comparing the Survey and Big Data

Results from both the social media data and the survey data indicate a mostly positive experience in the park. Even though there were some differences between users of the park and the respondents of the survey, their experiences in the park tended to be positive.

The results from the social media data and the survey data explained the users’ activities, satisfaction (sentiments), and their social interaction in the park. The survey data and the social media data represent the different aspects of the users’ activities. For example, the social media data could catch the extraordinary events of the daily lives of users such as eating delicious food with someone. The survey data included the ordinary, common activities in the park such as walking, relaxing, and chatting. In terms of social interaction, both methodologies support the idea that the park can contribute to social interaction. In terms of the social interactions, social media data and the survey data suggest that a park is a place to reinforce social interactions. According to the survey, users of the park indicated that the park contributed to social interaction and social cohesion. This result regarding the social interaction and satisfaction was identified as a positive relationship, however, the causal factors between two variables were not verified.

## 5. Conclusions

From the survey and social media, this study attempted to figure out the uses and meaning of urban parks. To overcome the problem that the survey may not reach sufficient users, social media can be used to fill a gap between a preset questionnaire and the reality. Through two lenses, the authors found meaningful implications. Through the survey, the results showed that park visitors mainly used the park for restoration and relaxation from their daily life. Additionally, park visitors who were more satisfied with their park experience also experienced more social cohesion and enhanced social interaction. Through the social media data, the authors found that social media users who visited and posted about the park enjoyed unique attributes or features of the park such as the railway, railroad, and forest features. Furthermore, social media users reacted positively to relatively small and common experiences such as coffee, beer, and casual foods in the park and negatively to less common and potentially threatening experiences such as a potential health and disease issues.

This study provides meaningful insights into the possible use of social media content as the data source for landscape architecture research. According to the study, the social media data and big data analytics can be used to detect a new activity type in a park. For the social interaction, park visitors mentioned ‘we’ when they posted positive contents instead of ‘I’. This may mean that social media users tend to share their status when they are with others.

This study also identified several limitations to the use of social media data in park studies, especially in terms of sampling challenges. By choosing a study site where young generations tend to visit, the authors tried to minimize a gap between social media users and real visitors. It may diffuse the challenges, but is not a perfect solution. The sampling challenges of social media data have to carefully consider when researchers use social media data for future research.

This study is a starting point in identifying more advanced research methods that can be used to augment existing methods. This paper makes several significant contributions to landscape architecture studies by providing a way to use social media data as a tool for understanding neighborhoods and resident preferences. Through the comparison of these methodologies, this study identified the pros and cons of the methods and explored the possibility of using big data analytics for understanding urban parks and the people who use them.

## Figures and Tables

**Table 1:** Data collection description.

Methods	Date	Sample Size	Respondents
Park visitor survey	08.15–08.18. 2018 10.01–10.08. 2018	177 ^1^	Park visitors (older than 18)
Social media data	06.10–09.20. 2018	3703	Keywords: ‘Gyeongui Line’, ‘Gyeongui Line Forest Park’ and ‘Yeontral Park’

^1^ Responses to the different methods of data collection.

**Table 2:** Characteristics of visitors.

Demographics	*n*	Percentage
Gender		
Male	81	45.8%
Female	96	54.2
Age		
18–29	64	36.2
30–39	50	28.3
40–49	33	18.6
50–59	21	11.9
Over 60	9	5.1
Residents ^1^		
Yes	54	30.5
No	123	69.5

^1^ Residents who live within 800 m of the park.

**Table 3:** Frequency of visit.

Numbers of Visit	Percentage
Every day	10.7%
More than 2 days a week	14.1
Once a week	9.6
1–3 times a month	11.9
Less than once a month	29.9
This is the first time	23.7

**Table 4:** Motivation and the actual activities in the park.

Activity	Stated Desire	Actual Use
Physical activities		
Biking	0.0%	0.0%
Walking or running	18.1	19.2
Mental health		
Refresh one’s mind	53.7	27.1
Relax and restoration	36.2	31.1
Social interaction		
Seeing others	26.6	41.7
Having time with friends or family	17.0	50.9
Other activities		
Just passing through the park	36.7	72.3
Enjoying hobby (photo, sports, etc.)	3.4	5.1
